# HNF4A expression as a potential diagnostic tool to discriminate primary gastric cancer from breast cancer metastasis in a Brazilian cohort

**DOI:** 10.1186/s13000-017-0635-2

**Published:** 2017-06-05

**Authors:** Patrícia Chaves de Freitas Campos Jucá, Stephany Corrêa, Giselle Maria Vignal, Maria Theresa de Souza Accioly, Suzana Angélica Silva Lustosa, Eliana Abdelhay, Delcio Matos

**Affiliations:** 1 Departamento de Câncer de Mama, HCIII, Instituto Nacional de Câncer (INCA), Rio de Janeiro, Brazil; 2 Instituto Nacional de Câncer (INCA), HCI, Centro de Transplante de Medula óssea (CEMO), Rio de Janeiro, Brazil; 3 Divisão de Patologia (DIPAT), Instituto Nacional de Câncer (INCA), Rio de Janeiro, Brazil; 4 Departamento de Cirurgia, Gastroenterologia Cirúrgica, Universidade Federal de São Paulo (UNIFESP), São Paulo, Brazil; 5 Instituto Nacional de Câncer (INCA), Hospital do Câncer III (HCIII) , Rio de Janeiro, Brazil

**Keywords:** HNF4A, Biomarker, Gastric cancer, Breast cancer, Metastasis

## Abstract

**Background:**

Among the many challenges in cancer diagnosis is the early distinction between metastatic cancer and a secondary tumor. This difficulty stems from the lack of markers that offer high sensitivity and specificity and can be easily applied in routine laboratory work. An example of this challenge is distinguishing gastric metastases originating from breast cancer from a gastric primary tumor. Hepatocyte nuclear factor 4 alpha (HNF4A) has been suggested as a potential marker in these cases.

The aim of this study was to analyze the expression of HNF4A, estrogen receptor (ER), progesterone receptor (PR) and gross cystic disease fluid protein 15 (GCDFP-15) in a Brazilian cohort.

**Methods:**

We performed immunohistochemistry analysis of HNF4A, ER, PR and GCDFP-15 in 126 patients divided into three cohorts: primary breast cancer, primary gastric cancer and both types of tumors.

**Results:**

Our data confirmed the sensitivity and specificity of the HNF4A marker compared to other currently used clinical markers.

**Conclusion:**

HNF4A alone could be a gold standard marker for distinguishing primary gastric cancer from breast metastasis, thus validating its potential clinical use, especially in populations with high genetic diversity.

**Electronic supplementary material:**

The online version of this article (doi:10.1186/s13000-017-0635-2) contains supplementary material, which is available to authorized users.

## Background

Breast cancer (BC) represents the major cause of cancer-related death among women. BC classification relies on several aspects of tumor biology, such as origin, tumor staging (tumor/node/metastasis (TMN) classification) and molecular expression [1, 2]. The most frequent histological subtypes of invasive carcinomas in BC are invasive ductal carcinoma (IDC) and invasive lobular carcinoma (ILC) [3]. Both types can metastasize to the gastrointestinal (GI) tract; however, the hematogenous dissemination patterns may differ between IDC and ILC, leading more commonly to GI tract metastasis from ILC tumors [4–9].

The accurate diagnosis of primary gastric cancer compared to gastric metastasis from BC can be difficult. Radiological, endoscopic and histopathological findings may be inconclusive. The clinical presentation of metastases to stomach resembles that of primary gastric adenocarcinoma; however, the prognosis and treatment substantially differ among patients who present with (1) both primary tumors (BC and gastric) and (2) primary BC and metastases to stomach.

Recently, two studies investigating a set of several markers to identify potential markers for discerning primary and metastatic gastric carcinoma [10, 11] highlighted HNF4A expression as a possible marker, but the analyzed test cohorts were small.

HNF4A is a nuclear transcription factor that binds DNA as a homodimer. The encoded protein has been suggested as a potential therapeutic target in early-stage gastric cancer [12]. HNF4A is correlated with invasion, metastasis and epithelial-to-mesenchymal transition [12, 13]. To demonstrate that HNF4A can serve as a biomarker, it is crucial to (1) increase the size of patient cohorts and (2) investigate populations with different genetic backgrounds.

Therefore, the aim of this study was to evaluate the capacity of a concise panel of known BC markers together with GCDFP-15 and HNF4A to accurately distinguish primary gastric adenocarcinoma from metastases to stomach in the Brazilian population. HNF4A expression, together with ER and PR expression, exhibited high sensitivity (100%) and specificity (96%) in the 126 patients analyzed in our study. Moreover, our data confirmed HNF4A as a potential marker in primary gastric adenocarcinoma and suggest the utility of a four-marker immunohistochemistry panel.

## Methods

### Patient recruitment

This retrospective study was performed at Instituto Nacional de Câncer (INCA) in collaboration with Escola Paulista de Medicina at Universidade Federal de São Paulo (UNIFESP). A total of 126 women were enrolled in this study. Patients were diagnosed with BC at INCA between January (1997) and March (2013), with follow-up until July (2014). The Reporting Recommendations for Tumor Marker Prognostic Studies (REMARK) criteria were followed for patient selection, assay performance, and data analysis throughout the study. The institutional board previously approved the procedures. The study was designed and conducted in accordance with ethical principles for medical research involving human subjects based on the Declaration of Helsinki. Approval was obtained from the Ethics Committee from INCA and UNIFESP institutes, with the following reference number: 12390213.3.00005274.

All diagnoses were made based on clinical, endoscopic (for gastric cancer) and histopathological data.

### Immunohistochemical experiments using tumor samples

Hematoxylin-eosin (HE) slides of each paraffin-embedded block of surgical specimens and biopsy samples were separated and analyzed by the pathologist to select slides with a higher content of tumor tissue and slides with normal tissue present as an internal control. Siliconized slides for immunohistochemical (IHC) analysis were produced by cutting ten histological sections to 3-μm thickness from each paraffin-embedded block. The following markers were used for IHC experiments in all patients: estrogen receptor (ER), progesterone receptor (PR), gross cystic disease fluid protein (GCDFP-15) and hepatocyte nuclear factor 4 alpha (HNF4A). As external positive controls, healthy breast tissue (for ER and PR), lymph nodal tissue (for GCDFP-15) and hepatic tissue (for HNF4A) were used.

Immunostaining with primary antibodies against ER (anti-human estrogen receptor α, clone 1D51 EgR at 1:1600; Dako), PR (anti-human progesterone, clone PgR 636 at 1:500; Dako), GCDFP-15 (clone 23A3 at 1:1000; Santa Cruz Biotechnology) and HNF4A (clone H1: sc-374229 at 1:100; Santa Cruz Biotechnology) was performed on all samples from patients with BC and/or gastric cancer.

The paraffin-embedded sections were heated for 30 min at 65°C, deparaffinized in xylene, and rehydrated using an ethanol gradient at room temperature. Incubations were performed in a humidified chamber. Sections were treated for 40 min at room temperature with 2% bovine serum albumin and were incubated overnight at 4°C with primary anti-human antibodies against ER, PR, GCDFP-15 and HNF4A. The secondary antibody provided in the commercial kit was added and incubated with the samples for 2 h. Horseradish peroxidase activity was visualized after treatment with H_2_O_2_ and 3,3′-diaminobenzidine (DAB) for 5 min (Novolink Polymer Detection Systems). In the last step, sections were weakly counterstained with Harry’s hematoxylin (Merck). The intensity and localization of the immunoreactivity were examined using a photomicroscope.

### Assessment of staining

Staining was interpreted by an expert pathologist (GMV) and the investigator (PJ) at the Pathology Division of the Instituto Nacional de Câncer (INCA). Samples with nuclear staining were considered positive for ER, PR and HNF4A, and samples with cytoplasmic staining were considered positive for GCDFP-15. Immunohistochemical expression was quantified according to the following criteria: negative expression, no staining signal; focal expression, positive staining of up to 1% of tumor cells; limited expression, positive staining of 10-50% of the cells; and diffuse expression, positive staining of more than 50% of the cells. To calculate the percentages for sensitivity and specificity, values were considered negative for cases with less than 1% expression and positive for cases with more than 1% expression.

### Patient cohorts

Patients were divided into three cohort groups (*n*=42 each) as follows: *control cohort I* comprised primary breast carcinoma patients with IDC or ILC and no other associated tumor; *control cohort II* included patients with primary gastric adenocarcinoma and no other associated tumor; and *test cohort III* included patients present with tumors in both breast and stomach. The *test cohort III* was subdivided into two subgroups (A) metastases to stomach derived from BC (*n*=25) and (B) primary breast carcinoma patients with IDC or ILC with primary gastric adenocarcinoma (*n*=17). This subdivision was based on positive results for the HR, GCDFP-15, CK 7 and CK 20 markers (found in 55% of the patients). The control cohorts were analyzed separately, but in the test cohort, only the stomach tissue was evaluated for diagnosis.

### Statistical analyses

For the statistical data analyses, SPSS, Excel and Project R software were used to calculate descriptive statistics of the variables and construct tables with diagnostic features of HNF4A, ER, PR and GCDFP-15 tests. Sensitivity refers to the ability of the test to classify an individual with the event. Specificity refers to the ability of the test to predict an individual with a non-event. Accuracy was calculated as the proportion of correct predictions made by using the markers [14]. Moreover, prevalence and predictive values and Fisher’s exact test were calculated to verify the association between the test results and the type of marker (used for each cohort) and the association between the test and the control cohort results (for each marker). Fisher’s exact test evaluates the null hypothesis of no association.

## Results

### Clinicopathological data

**Fig. 1:**
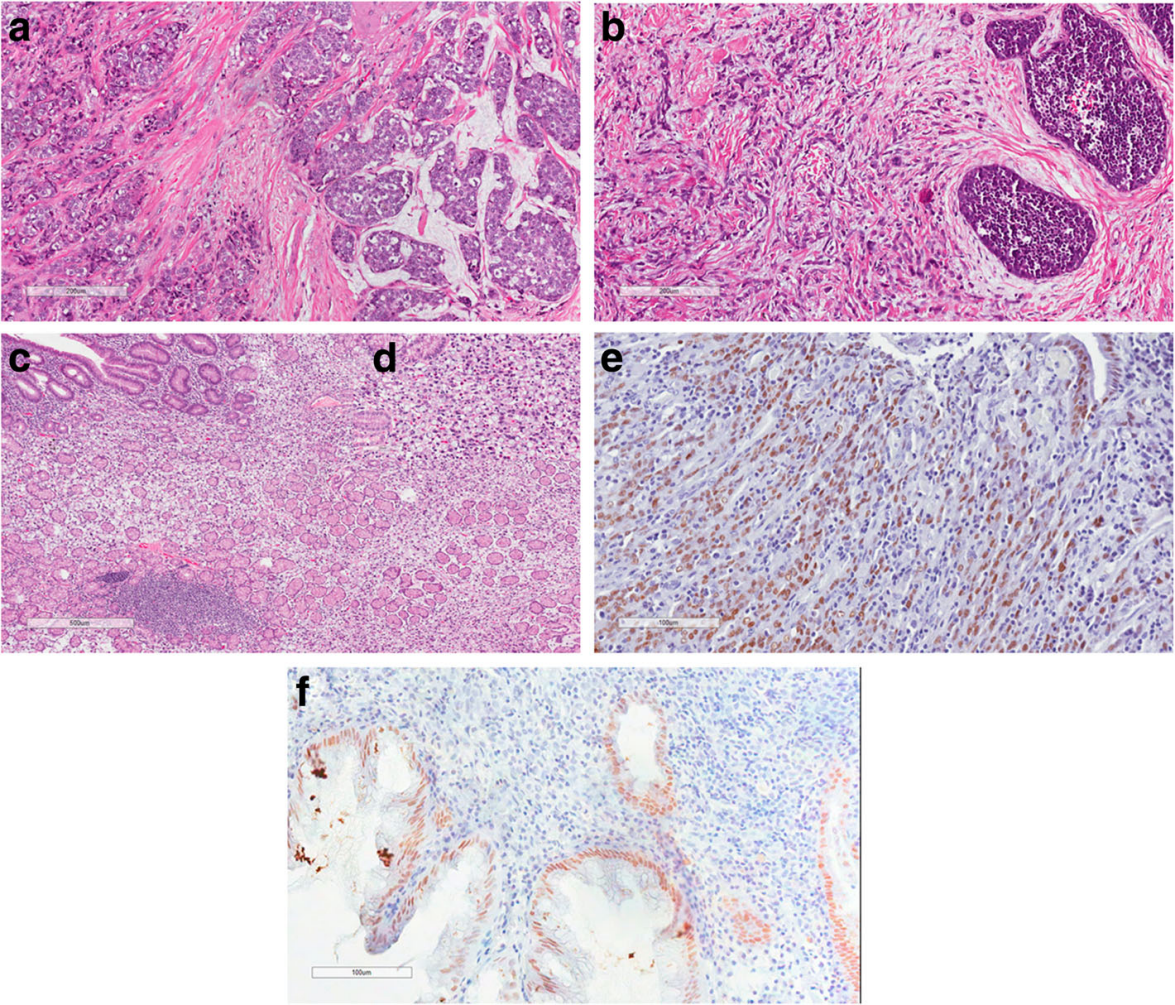
Representative immunohistochemistry results from the patient cohorts. **a** Invasive ductal carcinoma (IDC) (*left*) with mucin differentiation area (*right*). **b** Invasive lobular carcinoma (ILC) (*left*) associated with “*in situ*” lobular carcinoma (*right*). **c** Poorly differentiated primary gastric adenocarcinoma with signet ring cells (stomach). **d** Details at 20X magnification. **e** HNF4A staining in primary gastric adenocarcinoma. Nuclear staining in a non-neoplastic gland (*left*): internal control and nuclear staining in poorly differentiated gastric carcinoma cells with signet ring cells. **f** Metastasis from ILC to gastric mucosa. HNF4A nuclear staining in gastric cells from mucosa and non-staining from ILC cells

The clinicopathological data from patients showed that the mean age upon diagnosis was 54.2 years (range 38–87) for patients with primary breast carcinoma (cohort III-A) and 59.3 years (range 41–93) for patients with breast metastasis in the stomach. The mean interval between the diagnosis of primary breast carcinoma and the detection of gastric metastasis was 62 months (range 0–192 months). In four cases, gastric metastasis and primary breast carcinoma were diagnosed simultaneously. Interestingly, although ILC corresponds to a minor subset of BC cases, 13 of the patients (52%) in our study had ILC. The mean age at BC diagnosis was 57.3 years (range 34–73), and the mean age at primary gastric carcinoma diagnosis (cohort III-B) was 61.6 years (range 44–74). The disease-free interval between the diagnoses in the patients with two primary carcinomas was approximately 5 years. Three patients had gastric carcinoma as the primary tumor, and two patients had a concurrent diagnosis of breast and gastric cancers. In cohort III-B, of the 17 patients presenting with two primary carcinomas (both breast and gastric), 15 patients (88.2%) were diagnosed with IDC, and two were diagnosed with ILC. Patients in cohorts I and II had a mean age of 57.6 years (range 27–87) and 60.5 years (range 31–78), respectively, suggesting that there was no significant difference in age at diagnosis for the patients with primary tumors and those with cancer metastasis. The clinicopathological data for the patients were obtained from their medical records and are shown in Table 1. Representative pathology results for cohorts I, II and III are shown in Fig. 1.

**Table 1:** Patient clinicopathological data

Clinical data	Patients number (%)
Women	126
Age at diagnosis (range, years)	60.5 (27–93)
< 60 years	66 (52.4)
> 60 years	60 (47.6)
Primary Localization	
Breast	67 (53.2)
Stomach	59 (46.8)
Histological Type	
Primary gastric adenocarcinoma	42 (33.3)
Primary Breast Carcinoma	42 (33.3)
Gastric metastasis from Breast carcinoma	25 (19.9)
Primary gastric adenocarcinoma and primary breast carcinoma	17 (13.5)
Histological Grade	
I-II	50 (39.7)
III	76 (60.3)
Stage	
I	19 (15)
II	23 (18.3)
III	40 (31.8)
IV	43 (34.1)
Unkown	1 (0.8)
Mortality	
Alive	33 (26.2)
Dead	93 (73.8)
Follow up (range, months)	56.4 (1─186.4)

### ER, PR and GCDFP-15 immunohistochemical staining

To confirm the results obtained by pathological analysis, immunohistochemistry assays were conducted using known clinical markers: ER, PR and GCDFP-15. First, the three cohort groups were analyzed separately for each marker.

Regarding ER expression, of the 25 patients in cohort III-A, 12 patients (48%) were positive for gastric tumors. Additionally, of the 42 patients in cohort I, 35 (83%) were positive for ER. No staining was observed in cohort II or cohort III-B. These results are shown in Table 2. For the expression of PR, 6 (24%) and 31 (73.8%) patients were positive in cohorts III-A and I, respectively. Similar to ER expression, no staining was observed in either cohort II or III-B. These results are shown in Table 3. GCDFP-15 immunostaining was positive in 9 (36%) patients from cohort III-A and 15 (35.7%) patients from cohort I. Again, no staining was observed in either cohort II or cohort III-B. These results are shown in Table 4.

**Table 2:** Distribution of ER expression by number and percentage in cohorts I, II and III

	Histological type of cancer					
	Test		Control		Total	*P*
	Cohort III-A	Cohort III-B	Cohort I	Cohort II	*n* (%)	
	*n* (%)	*n* (%)	*n* (%)	*n* (%)		
Negative	13 (52.0)	17 (100.0)	7 (16.4)	42 (100.0)	79 (62.7)	<0.0001
Positive	12 (48.0)	0 (0)	35 (83.3)	0 (0)	47 (37.3)	
Total	25 (100.0)	17 (100.0)	42 (100.0)	42 (100.0)	126 (100.0)

**Table 3:** Distribution of PR expression by number and percentage in cohorts I, II and III

	Histological type of cancer					
	Test		Control		Total	*P*
	Cohort III-A	Cohort III-B	Cohort I	Cohort II	*n* (%)	
	*n* (%)	*n* (%)	*n* (%)	*n* (%)		
Negative	19 (76.0)	17 (100.0)	11 (26.2)	42 (100.0)	89 (70.6)	<0.0001
Positive	6 (24.0)	0 (0)	31 (73.8)	0 (0)	37 (29.4)	
Total	25 (19.8)	17 (13.5)	42 (33.33)	42 (33.33)	126 (100.0)

**Table 4:** Distribution of GCDFP-15 expression by number and percentage in cohorts I, II and III

	Histological type of cancer					
	Test		Control		Total	*p*
	Cohort III-A	Cohort III-B	Cohort I	Cohort II	*n* (%)	
	*n* (%)	*n* (%)	*n* (%)	*n* (%)		
Negative	16 (64.0)	17 (100.0)	27 (64.3)	42 (100.0)	102 (81.0)	<0.0001
Positive	9 (36.0)	0 (0)	15 (35.7)	0 (0)	24 (19.0)	
Total	25 (19.8)	17 (13.5)	42 (33.33)	42 (33.33)	126 (100.0)	

### HNF4A staining and marker-panel construction

Because HNF4A has been reported as a relevant marker for discriminating primary and metastatic gastric carcinoma, we evaluated its expression alone and within two panels (with or without HNF4A expression analysis). In all positive cases, the expression of HNF4A was greater than 40%. For HNF4A expression alone, the 17 patients previously diagnosed with primary adenocarcinoma (cohort III-B) exhibited positive immunohistochemical staining, thus confirming their primary status. Positive immunostaining for this marker was also observed in one patient from cohort III-A who had been previously diagnosed with metastatic cancer. HNF4A showed positive expression in all patients diagnosed with primary adenocarcinoma (cohort II) and was negative in all cases of primary breast carcinoma (cohort I). These results are shown in Table 5.

**Table 5:** Distribution of HNF4A expression by number and percentage in cohorts I, II and III

	Histological type of cancer					
	Test		Control		Total	*p*
	Cohort III-A	Cohort III-B	Cohort I	Cohort II	*n* (%*)*	
	*n* (%)	*n* (%)	*n* (%)	*n* (%)		
Negative	24 (96.0)	0 (0)	42 (100.0)	0 (0)	66 (52.4)	<0.001
Positive	1 (4.0)	17 (100.0)	0 (0)	42 (100.0)	60 (47.6)	
Total	25 (100.0)	17 (100.0)	42 (100.0)	42 (100.0)	126 (100.0)	

To evaluate the diagnostic value (*test*) of each marker in cohort III, the sensitivity, specificity, positive predictive value, and negative predictive value (NPV) for breast and gastric cancer were considered, as was the total accuracy of each marker. As shown in Additional file 1, the sensitivity for the ER-PR-GCDFP-15 panel was 52%; however, when combined with the HNF4A marker, the sensitivity reached 100.0%. Specificity was slightly higher for the ER-PR-GCDFP-15 panel (100.0%) than for the HNF4A marker alone (96.0%). Specificity and NPV could not be evaluated for all four markers because there was no HNF4A staining in BC (cohort I).

In general, including the HNF4A marker positively increased the evaluated indicators and enhanced the discrimination of primary gastric cancer and metastatic gastric cancer.

## Discussion

One important area in the study of cancer is the origin of the tumor. There are important differences in tumor biology between a primary tumor and a metastatic tumor at the same site, and those peculiarities define the prognosis and therapeutic approach.

An association has been reported to exist between BC and gastric cancer, with the latter as a possible second primary tumor or a metastasis site. For GIT metastasis originating from primary BC, the stomach is one of the most frequently affected organs [15]. Therefore, the discrimination of both tumors is essential to determining a patient’s clinical course and treatment outcome.

For this purpose, the expression of markers such as ER, PR, and GCDFP-15 is evaluated in conjunction with pathology analyses. However, these markers may lack sensitivity and/or specificity. Recent studies have indicated that HNF4A expression is a powerful marker for distinguishing a gastric primary tumor from BC metastasis. Koyama and colleagues demonstrated that HNF4A could distinguish all primary gastric carcinomas from metastatic breast carcinomas, suggesting that HNF4A may be a highly useful marker for excluding metastatic breast carcinoma in the diagnosis of gastric specimens [10]. Post and colleagues showed that HNF4A is a highly useful marker for discriminating primary and metastatic breast carcinomas from gastric carcinomas, with a sensitivity of 99% and specificity of 100% [11], thus supporting the data of Koyama and colleagues; however, both studies examined only a few patients.

In the present work, we aimed to evaluate HNF4A together with the expression of ER, PR, and GCDFP-15 to investigate the discrimination of cancers in 126 Brazilian patients.

Our results for the ER, PR and GCDFP-15 expression panel are consistent with literature data, showing that the evaluation of these proteins is specific but not sufficiently sensitive to discriminate BC metastasis in the stomach from primary gastric cancer. The analysis of HNF4A in conjunction with the previous panel clearly increased the diagnostic value and supported the literature data. HNF4A showed no expression in the stomach tissue in 24 patients in cohort III-A, thus confirming the mammary origin of the disease. One case presented expression of HNF4A, together with negative expression for ER, PR and GCDFP-15. This observation raised the hypothesis that this patient could have been misdiagnosed, based on pathology analysis alone.

It has been discussed, in literature, that GI metastasis from the breast vary from 0.1 to 0.3% in retrospective series and up to 10% from autopsia series, given the difficulty in diagnose [10]. Although ILC accounts for only 5% to 10% of invasive breast lesions, most GI metastasis from the breast originates from ILC [16]. In this study, 40% of gastric metastasis was of ILC origin, a high percentage compared to previous studies but still in agreement with the data reported more recently in the literature [17]. However, 60% of gastric metastasis was of IDC origin. In addition to sharing many clinical, radiological and endoscopic features with primary gastric carcinoma, metastasis may present with signet ring cells, increasing the difficulty of differentiation. Signet cells were observed in several of our patients, all of whom had gastric metastasis originating from IDC (10 of 15), ratifying that such feature is not exclusive from ILC tumors.

## Conclusion

Although large prospective studies are needed to determine whether HNF4A alone represents a gold standard marker for distinguishing primary gastric cancer from breast metastasis, our data from a Brazilian cohort provide substantial insight into the use of this marker, consistent with the existing literature data, and evidence regarding the genetic diversity of the Brazilian population.

## Additional file

Additional file 1: Distribution of the sensitivity, specificity, positive predictive value, negative predictive value and accuracy percentage values for the ER, PR, GCDFP-15 and HNF4A markers. (XLS 36 kb)

## Abbreviations

BC: Breast cancer
CK: Cytokeratin
DAB: 3,3′-diaminobenzidin
ER: Estrogen receptor
GCDFP-15: Gross cystic disease fluid protein
GI: Gastrointestinal
HE: Hematoxylin-eosin
HNF4A: Hepatocyte nuclear factor 4 alpha
IDC: Invasive ductal carcinoma
IHC: Immunohistochemical
ILC: Invasive lobular carcinoma
INCA: Instituto Nacional de Câncer
PR: Progesterone receptor
REMARK: Reporting recommendations for tumor marker prognostic studies
TMN: Tumor/node/metastasis
UNIFESP: Universidade Federal de São Paulo
